# TSPO Radioligands for Neuroinflammation: An Overview

**DOI:** 10.3390/molecules29174212

**Published:** 2024-09-05

**Authors:** Silvia Salerno, Monica Viviano, Emma Baglini, Valeria Poggetti, Doralice Giorgini, Jacopo Castagnoli, Elisabetta Barresi, Sabrina Castellano, Federico Da Settimo, Sabrina Taliani

**Affiliations:** 1 Department of Pharmacy, University of Pisa, Via Bonanno 6, 56126 Pisa, Italy; silvia.salerno@unipi.it (S.S.); emma.baglini@farm.unipi.it (E.B.); valeria.poggetti@phd.unipi.it (V.P.); jacopo.castagnoli@phd.unipi.it (J.C.); sabrina.taliani@unipi.it (S.T.); 2 Department of Pharmacy, University of Salerno, Via Giovanni Paolo II, 132, 84084 Fisciano, Italy; mviviano@unisa.it (M.V.); dgiorgini@unisa.it (D.G.); scastellano@unisa.it (S.C.)

**Keywords:** translocator protein, diagnostic marker, radioligand, neuroinflammation

## Abstract

The translocator protein (TSPO) is predominately localized on the outer mitochondrial membrane in steroidogenic cells. In the brain, TSPO expression, low under normal conditions, results upregulated in response to glial cell activation, that occurs in neuroinflammation. As a consequence, TSPO has been extensively studied as a biomarker of such conditions by means of TSPO-targeted radiotracers. Although [^11^C]-PK11195, the prototypical TSPO radioligand, is still widely used for in vivo studies, it is endowed with severe limitations, mainly low sensitivity and poor amenability to quantification. Consequently, several efforts have been focused on the design of new radiotracers for the in vivo imaging of TSPO. The present review will provide an outlook on the latest advances in TSPO radioligands for neuroinflammation imaging. The final goal is to pave the way for (radio)chemists in the future design and development of novel effective and sensitive radiopharmaceuticals targeting TSPO.

## 1. Introduction

Neuroinflammation is an inflammatory and adaptive response within central nervous system (CNS), which includes the brain and spinal cord. The correct functionality of the brain can be negatively affected by different external agents, including injury, ischemia, infection, toxins, neurotoxic stimuli, autoimmunity and responses to processes that may change neuronal activity. As a consequence, cellular and biochemical responses are generated by the brain cells, predominantly microglia and astrocytes, by activation of what is called “acute neuroinflammation”. This process implies the disruption of toxic agents and irreversible damaged tissue, the protection of surrounding cells and the repairment of tissue, limiting the spread of damage [1]. The neuroinflammatory response aims to mitigate the activation of factors by evoking CNS immunity to defend against potential damage and to maintain and restore homeostasis.

Therefore, neuroinflammation represents a physiological process crucial for the protection of the CNS. However, if inflammation becomes chronic, it can cause damage to healthy brain tissue and contribute to the development of neurological diseases, including Alzheimer’s disease (AD), Parkinson’s disease (PD), and multiple sclerosis (MS) [2,3].

In this scenario, understanding the mechanisms underlying neuroinflammation is crucial for developing targeted treatments that can regulate inflammation and shield the brain from the adverse effects of excessive or abnormal inflammatory responses.

Interestingly, neuroinflammation seems to be correlated with the overexpression of the translocator protein (TSPO). Therefore, it may be considered a suitable neuroinflammation biomarker as well as a target for positron emission tomography (PET) and single-photon emission computed tomography (SPECT) imaging [4,5,6].

**Figure 1:**
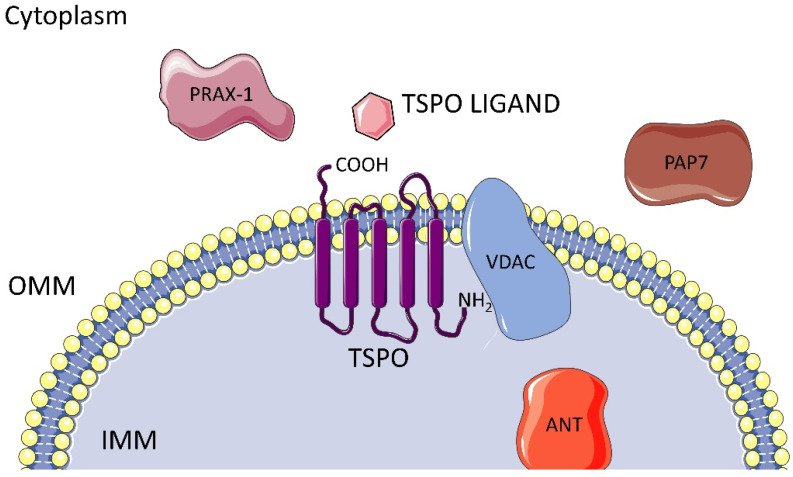
TSPO localization and associated proteins.

TSPO (18 kDa) is an evolutionarily well-conserved and tryptophan-rich 169-amino-acid protein with 5 trans-membrane domains, each comprised of 21 residues that spread across the whole membrane, with the carboxyl portion outside and the amino one inside the mitochondria. It is predominantly localized at the contact sites between the outer (OMM) and the inner mitochondrial membrane (IMM), where it plays a key role in the translocation of cholesterol, namely the rate-limiting step of steroidogenesis, Figure 1. Furthermore, TSPO actively participates in various physiological processes beyond its primary function, including immunomodulation, mitochondrial metabolism and function. TSPO is also involved in apoptosis, cell respiration, oxidation, cell proliferation and differentiation. Additionally, it contributes to porphyrin transport, heme biosynthesis and protein and ion transport [7,8,9,10].

TSPO is associated with the voltage-dependent anion channel (VDAC), the adenine nucleotide translocase (ANT), and other mitochondrial proteins like PRAX-1, PAP7, hence forming the mitochondrial permeability transition pore (MPTP), Figure 1.

Recently, researchers employed different methods to obtain structural insights into TSPO. Jaremko et al. used NMR spectroscopy to determine the high-resolution three-dimensional structure of mouse TSPO (mTSPO) in complex with its high-affinity ligand PK11195 [11,12]. Findings revealed that PK11195 binds to a hydrophobic crevice within the helical bundle of TSPO. Of note, mTSPO structure appeared highly stable when complexed with PK11195. However, subsequent analyses evidenced that the absence of a high-affinity ligand confer a certain conformational flexibility in the mTSPO structure [13].

Shortly after this study, X-ray crystallography was employed to elucidate the crystal structure of TSPO homologues from two bacterial species, namely *Rhodobacter sphaeroides* (*Rs*TSPO) [14] and *Bacillus cereus* (*Bc*TSPO) [15]. These investigations proved that monomeric TSPO molecules form dimers within the membrane and that each monomer comprises five transmembrane helices.

Under healthy physiological conditions, TSPO is extensively diffused across various peripheral organs, such as the heart, lungs, kidney, adrenal glands, and nasal epithelium. Steroid-producing tissues exhibit the highest concentration of TSPO, while reduced levels are noted in the liver and in quiescent microglial cells and neurons in the normal brain.

The TSPO expression is altered in a wide range of human diseases [16]. Furthermore, TSPO overexpression has been found in certain cancerous conditions, including breast [17] and colorectal cancer [18], glioma [19] and hepatocellular carcinoma [20]. It has been suggested that TSPO levels may be correlated with the metastatic potential of breast cancer and gliomas. Conversely, reduced levels of TSPO have been identified in patients with generalized anxiety, schizophrenia, panic disorder, post-traumatic stress disorder, and obsessive-compulsive disorder [18]. In the CNS, it is predominantly upregulated in microglia in response to inflammatory stimuli. TSPO overexpression has also been observed in reactive astrocytes. Increased levels of TSPO in glial cells have been associated with several neurodegenerative and neuroinflammatory disorders, including PD, Huntington’s disease, MS, amyotrophic lateral sclerosis, AD, and certain psychiatric conditions [16,21].

Although in vivo structural imaging offers valuable data in preclinical and clinical studies, it is essential to combine morphological information and in vivo molecular imaging to unveil the real-time structures of dynamic physiological processes underlying disease phenomena. Among the tomographic molecular imaging modalities, PET imaging provides more translational possibilities compared to others thanks to its sensitivity combined with its quantitative accuracy. PET is a noninvasive imaging technique offering physiological information by means of radiotracer injection, detection of its radiation, and reconstruction of its distribution. Thus, PET is nowadays a standard diagnosis imaging technique in oncological, neurological, and cardiovascular diseases. This is principally attributed to the clinically useful information it offers about organ and tissue function and status, by using radiolabeled molecular imaging agents. Depending on imaging agent and disease, the type of information obtained may include detection, classification, staging, prognosis, treatment planning, assessing of response to therapy, and surveillance [22].

The radiotracer [^11^C]PK11195 (**1**) has been extensively utilized in PET imaging to study neuroinflammation. One of the notable advantages of [^11^C]PK11195 (**1**) is its limited susceptibility to a specific TSPO genetic variation known as a single nucleotide polymorphism (SNP) rs6971 in humans. The allelic frequency of the SNP rs6971 is approximately 30% in individuals of European ancestry. This SNP, lacking known clinical significance, exhibits codominant expression, resulting in the formation of high-affinity binders (HABs with two high-affinity alleles), low-affinity binders (LABs with two low-affinity alleles), and mixed-affinity binders (MABs with one low and one high-affinity allele). HABs are homozygous for wild-type TSPO, whereas MABs are heterozygous and LABs are homozygous for the Ala147Thr TSPO [23]. Thus, individuals with the same TSPO density but different genotypes will produce different PET signals. The difference in affinity between the two alleles can be significant for certain radioligands. The relevance of the SNP rs6971 in TSPO to the interpretation of clinical PET data is evident. Although each radioligand has a unique level of sensitivity, studies with TSPO radioligands must take SNP rs6971 into account by doing TSPO genotyping before imaging. This task is an unwelcome extra burden in any PET study. Often, LABs must be excluded because the brain has too little uptake to quantify and studies that include HABs and MABs need a suitable correction for binding levels across genetic groups [24,25,26].

However, [^11^C]PK11195 (**1**) presents some drawbacks, such as a high level of nonspecific binding [27,28] and a non-optimal signal-to-noise ratio compared to that in the early years of TSPO PET imaging, complicating its quantification [29]. Although quantification problems have been circumvented [30,31], there is still a need for the development of new TSPO radioligands that can offer improved performance in quantifying TSPO expression while still maintaining low sensitivity to the SNP rs6971 [6].

With respect to our previous overview published in 2022 [32], in this review we make an update on the recent advances in the field of TSPO radioligands for neuroinflammation elucidating concerns with regard to the imaging of TSPO with PET taking into particular account the impact of the SNP rs6971 on second-generation ligand binding, that have permitted to obtain higher TSPO PET brain signal in neuroinflammation diseases compared to control [33], thus suggesting that TSPO is a good target for development of neuroinflammation imaging agents. Furthermore, we give more information about current clinical trials concerning TSPO radioligands for neuroinflammation and we have expanded and updated the section relative to the development of third-generation TSPO radioligands that combine the TSPO-specific signals seen in second-generation ligands with a reduced sensitivity to SNP.

The availability of structural information on mTSPO in complex with its high-affinity ligand (*R*)-PK11195 by means of NMR spectroscopy [11,12], and the X-ray resolution of the crystal structures of TSPO homologues from two bacteria *Rs*TSPO [14] and *Bc*TSPO [15], together with the most recent advancement in TSPO radioligands for neuroinflammation imaging herein comprehensively reviewed, will help (radio)chemists in the design and development of increasingly specific and effective radiopharmaceuticals targeting TSPO, hopefully leveraging the recent efforts in TSPO radioligand design focusing on the development of ligands with low SNP rs6971 sensitivity and thus definitively cutting out the extra burden in performing TSPO genotyping before any PET study.

## 2. TSPO Radioligands

The development of radiotracers for PET/SPECT imaging of molecular targets in the CNS is challenging, particularly with regard to quantitation aspects. The first issue to be considered concerns the ability of the radiotracer to cross the blood–brain barrier (BBB). Generally, passive transfer across the BBB is facilitated by low molecular weight (<500 Da), lack of formal charge, low hydrogen-binding capability, and modest lipophilicity (Log*D* between 2.0 and 3.5) [34]. Once within the CNS, an efficient radioligand has to bind with high affinity and selectivity to its target of interest, which should be of sufficient abundance for detection against a background of nonspecific binding [35].

For reversible radiotracers, specific binding increases over time until reaching an equilibrium defined by B_max_ (maximum number of binding sites) and K_d_ (tracer concentration that binds to half the binding sites at equilibrium) [36]. The ratio B_max_/K_d_ represents non-displaceable binding potential (BP_ND_), which reflects the specific binding. In particular, BP_ND_ less than 1 means low specific signal, while that exceeding 10 means a failure in reaching equilibrium within the time constraints of a dynamic PET scan. In both cases, quantitation becomes challenging.

Two radionuclides are commonly used to label TSPO PET radioligands, that are carbon-11 (^11^C) and fluorine-18 (^18^F). The relevant feature of ^18^F is its longer half-life (t_1/2_ = 109.8 min) relative to that of ^11^C (t_1/2_ = 20.4 min). In addition, PET images with a certain higher spatial resolution can be generated with fluorine-18 than with carbon-11, due to its lower positron energy [37,38]. The widespread occurrence of carbon in natural products and pharmaceuticals makes carbon-11 a valuable and appealing positron-emitting isotope for labeling biologically significant molecules. A major advantage is that ^11^C-labeled molecules exhibit the same chemical and biological behavior as their unlabeled counterparts. This is crucial as it eliminates concerns about the potential impact of adding an “artificial” PET tag (such as incorporating an ^18^F atom; *vide infra*) on the biological properties of the compound of interest. Almost all TSPO radioligands developed so far have a tertiary *N*-methyl amide position that is usually readily labeled by ^11^C-methylation. Carbon-11, due to its short half-life, cannot be transported to remote facilities, and the final radiotracer must be produced near the synthesis site. However, an advantage of carbon-11 for local use is that a subject can be injected multiple times on the same day with the same radioligand, and this may be useful for conducting a baseline experiment followed by an experiment in which the same subject undergoes a pharmacological or other type of challenge. In general, the rapid decay of carbon-11 means that the radiation exposure to the subject is well within approved limits. Fluorine-18 is frequently called the “radionuclide of choice” in PET imaging due to its advantageous physical and nuclear properties: a short yet manageable half-life of 110 min, which provides enough time for multistep synthetic labeling reactions, and a short positron linear range in tissue (2.3 mm) that produces the highest resolution PET images among all available positron emitters. The half-life of the ^18^F isotope is also sufficiently long to enable the transportation of doses to locations several hours away from the site of production. Even though fluorine is typically absent in a TSPO ligand, it can often be inserted at a hydrogen position in many ligands, one of the most common bioisosteric replacements in medicinal chemistry. Replacing hydrogen with fluorine in an organic molecule causes minimal steric disruption due to their similar van der Waals radii (1.35 and 1.20 Å, respectively), but it significantly increases the molecular weight. The strong electron-withdrawing nature of fluorine, as evidenced by the dipole moment of the carbon-fluorine bond, can alter the electronic properties and thus the biological behavior of the molecule. Substituting a hydrogen atom with fluorine is expected to modestly increase the lipophilicity of a molecule, based on the π coefficient of 0.14 for fluorine. However, fluorination does not always lead to an increase in lipophilicity; this bioisosteric substitution can reduce overall lipophilicity in certain structural contexts. Therefore, labeling with fluorine-18 usually results in modifications to the physicochemical properties and biological activity of known ligands. Additionally, replacing hydrogen with fluorine is a well-established approach in medicinal chemistry to modulate metabolism, as the electron-withdrawing properties of fluorine and the high strength of the carbon-fluorine bond make it chemically resistant under most biological conditions. Nonetheless, nearly all PET radioligands undergo significant metabolism during the course of a PET scan, sometimes generating radiometabolites that can cross the BBB and affect brain radioligand quantification. It is important to note that ^18^F-labeled ligands may be subject to peripheral radiodefluorination, resulting in [^18^F]fluoride ions in plasma that can be taken up by bone. If the radioactivity in the skull becomes significant, the accurate measurement of radioactivity in brain regions near the skull, such as the neocortex, will be compromised. Although it is difficult to predict the susceptibility of an ^18^F-labeled compound to radiodefluorination in human subjects, fluorine-18 atoms bound to benzene or pyridine rings are generally resistant to metabolic defluorination. Radioligands labeled with fluorine-18 in an aryl methoxy group are known to be susceptible to radiodefluorination, while fluorine-18 at the terminus of a straight saturated alkyl chain of two or more carbons is usually more resistant to defluorination in human subjects. In the development of TSPO radioligands, the preparation of ^18^F-labeled ligands is often carried out alongside the synthesis of ^11^C-labeled analogues. Fluorine-18 is frequently incorporated into a benzene ring, an *N*-methyl or *N*-ethyl position, or at the end of aryl fluoroalkoxy groups [37,38].

**Figure 2:**
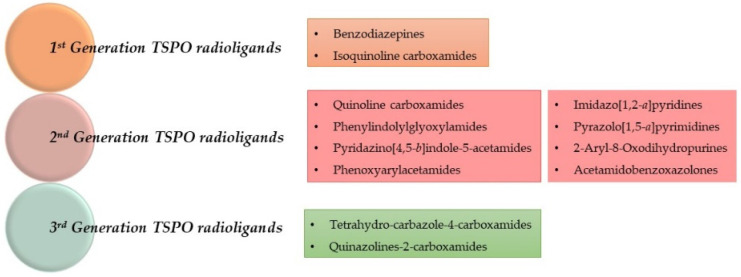
Classification of TSPO radioligands.

**Figure 3:**
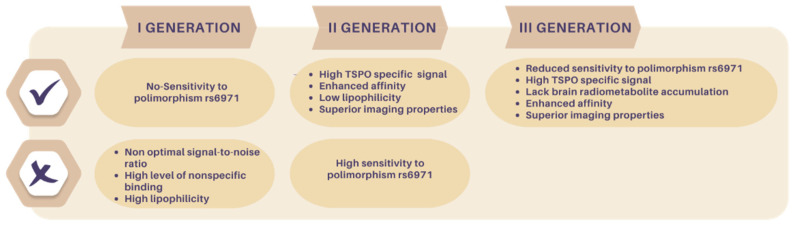
Advantages and disadvantages of first, second, and third generation of TSPO radioligands.

TSPO radioligands are categorized in three generations, as depicted in Figure 2 and Figure 3 and in Table 1, which lists the most representative compounds implicated in PET neuroinflammation studies.

**Figure 4:**
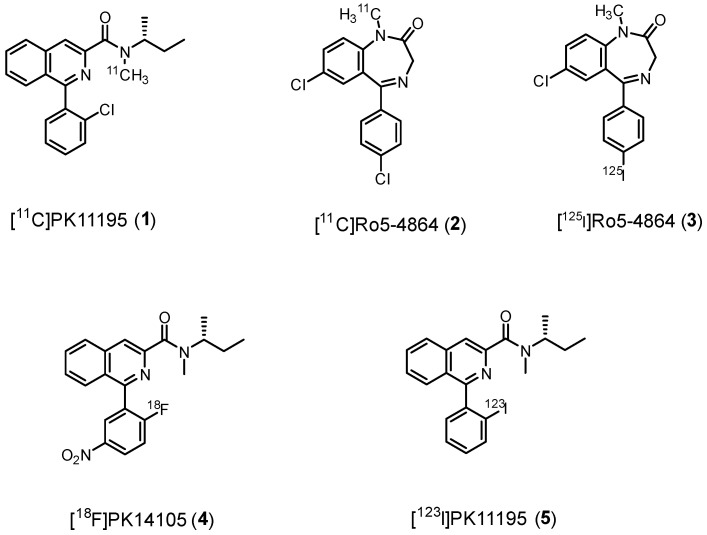
First-generation TSPO radioligands.

First-generation TSPO radiotracers include isoquinoline carboxamides, like [^11^C]PK11195 (**1**), and benzodiazepines, like [^11^C]Ro5-4864 (**2**), Figure 4.

Second-generation TSPO PET radiotracers comprise those compounds developed to overcome the abovementioned [^11^C]PK11195 (**1**) limitations. These radioligands have emerged from diverse structural classes and exhibit enhanced affinity and reduced lipophilicity compared to **1**, Figure 3. As a result, they demonstrate improved TSPO-specific signals and superior imaging properties. However, at variance with **1**, these radioligands suffer from sensitivity to SNP rs6971, Figure 3.

The development of third-generation TSPO radioligands aimed to combine the TSPO-specific signals seen in second-generation ligands with the reduced sensitivity to SNP exhibited by **1** (Figure 3).

### 2.1. First Generation TSPO Radioligands

#### 2.1.1. Benzodiazepines

Ro5-4864, (7-chloro-5-(4-chlorophenyl)-1-methyl-1,3-dihydro-2*H*-benzo[*e*][1,4]diazepin-2-one, Figure 4, Table 1) presents nanomolar affinity for TSPO (*K*_i_ = 6.0 nM) and a 1000-fold lower affinity for central benzodiazepine receptor (CBR). For this reason, it was radiolabeled for imaging purposes. However, early PET studies using [^11^C]Ro5-4864 (**2**) in humans with gliomas failed to demonstrate elevated radioactivity levels compared to healthy brain regions. Similarly, in vitro studies showed minimal binding of **2** in surgically removed human glioma samples [40]. These disappointing results could be attributed to various factors, such as elevated non-specific binding, high lipophilicity, low brain uptake, as well as species-specific binding [72].

Chlorine was replaced with iodine-125 to give [^125^I]Ro5-4864 (**3**), but unfortunately, no successful SPECT imaging was obtained. As a result of these challenges and the discovery of the isoquinolinecarboxamide class of compounds, which proved to be more effective TSPO ligands, benzodiazepines like Ro5-4864 have been deemed unsuitable for further development as tools for TSPO PET imaging.

#### 2.1.2. Isoquinoline Carboxamides

A few years earlier than **2**, [^11^C]PK11195 (**1**) (1-[2-chlorophenyl]-*N*-methyl-*N*-[1-methyl-propyl]-3-isoquinoline carboxamide (Figure 4, Table 1) was synthesized [29] and studied, quickly demonstrating its superiority with respect to **2**; **1** exhibited greater overall uptake and higher specific binding in gliomas with respect to **2** [40]. Initially, it was used as a racemate (*K*_i_ = 9.3 nM), but subsequent in vivo studies revealed that the (*R*)-enantiomer showed significantly higher retention in abundant TSPO binding sites. This correlated with in vitro experiments, which showed the (*R*)-enantiomer to have 2-fold higher affinity for TSPO compared to the (*S*)-enantiomer (Table 1) [39].

As the prototypical first-generation TSPO radioligand, **1** was widely employed for decades in imaging TSPO in neurological and psychiatric disorders. For instance, it was used in clinical trials to investigate the role of activated microglia in several conditions, including cognitive impairment, psychosis/Schizophrenia, depression, addiction amyotrophic lateral sclerosis, complex regional pain syndrome, Creutzfeldt–Jakob disease, and other neurodegenerative diseases such as synucleinopathies and tauopathies, but also in viral infections, vascular diseases, traumatic brain injury, etc. For a comprehensive review, see Ref. [73]. AD, together with its prodromal condition, mild cognitive impairment (MCI), is the most studied pathology and one of the first pathologies studied apart from glioma, with [^11^C]PK11195 and (*R*)-[^11^C]PK11195 observed in patients since 1995 [74,75,76,77,78].

Despite being extensively used, **1** presents limitations that hinder its suitability as a PET tracer. One major concern is its relatively high lipophilicity (log*D* = 3.97), not in the ideal range (Log*D* range = 2.0–3.5) [34,79], leading to significant nonspecific binding, which, together with modest affinity, leads to a poor signal-to-noise ratio. Additionally, high plasma protein binding and low brain permeability further hinder its quantification [80,81]. These factors limit its capability to detect slight changes in TSPO density, making it unsuitable for nuclear imaging in certain diseases [82].

Nevertheless, **1** displays notably low SNP rs6971 sensitivity. It binds with similar affinity in all subjects, which may explain why non-binding has not been reported with this radioligand. Actually, this phenomenon of non-binding may have been overlooked with [^11^C]-(*R*)-PK11195 because this radioligand has a low ratio of specific to non-specific binding in the brain. The high non-specific binding of [^11^C]-(*R*)-PK11195 may have obscured real differences of specific binding in binders compared to non-binders [27,83].

Later, PK11195-derivatives labeled with fluorine-18, (*N*-methyl-*N*-(1-methylpropyl)-1-(2-fluoro-5-nitrophenyl)isoquinoline-3-carboxamide, [^18^F]PK14105 (**4**)) [84] or iodine-123, (1-(2-iodophenyl)-*N*-methyl-*N*-(1-methylpropyl)isoquinoline-3-carboxamide, [^123^I]PK11195, (**5**)) [85] were also synthesized, Figure 4. However, both radioligands displayed a decrease in in vitro affinity compared to **1** and no advantages initially in in vivo studies, rendering such compounds unworthy of further study.

### 2.2. Second Generation TSPO Radioligands

#### 2.2.1. Quinoline-2-carboxamides

**Figure 5:**
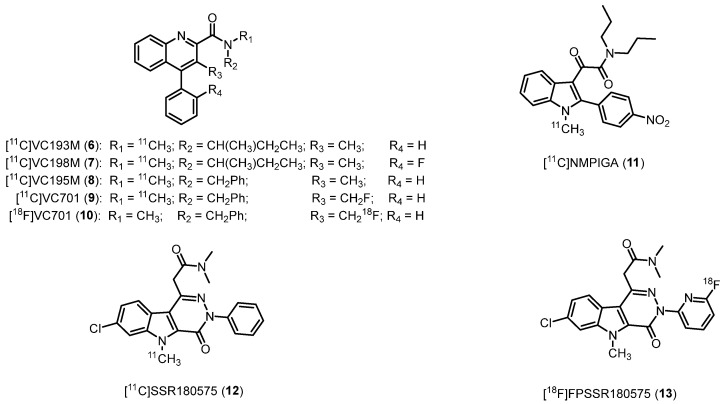
Second-generation TSPO radioligands.

This class of ligands was designed as conformationally restrained analogues of **1** [86]. The most potent representatives as TSPO probes are radiolabeled with carbon-11, (*N*-(*sec*-butyl)-*N*,3-dimethyl-4-phenylquinoline-2-carboxamide, [^11^C]VC193M (**6**), IC_50_ = 2.1 nM), (*N*-(*sec*-butyl)-4-(2-fluorophenyl)-*N*,3-dimethylquinoline-2-carboxamide, [^11^C]VC198M (**7**), IC_50_ = 2.9 nM), and (*N*-benzyl-*N*,3-dimethyl-4-phenylquinoline-2-carboxamide, [^11^C]VC195 (**8**), IC_50_ = 2.1 nM), Figure 5, Table 1 [41]. Initially, these derivatives were assessed for their ability to image microglia activation in neurodegenerative conditions [87].

Among them, the most interesting compound resulted **8**, which exhibited results similar to those of **1**. Subsequently, the fluoromethyl analogue [^11^C]VC701 (**9**) (*N*-benzyl-3-(fluoromethyl)-*N*-methyl-4-phenylquinoline-2-carboxamide, Figure 5, Table 1) was synthesized. It displayed an IC_50_ of 0.11 nM for TSPO, being approximately 20 times more potent with respect to **1**. In biodistribution experiments, **9** displayed higher tissue-to-plasma ratios than **8**, indicating reduced interaction with plasma proteins [42]. Additionally, VC701 was radiolabeled with fluorine-18 ([^18^F]VC701, **10**, Figure 5) to evaluate its kinetics and pharmacological properties in animals, where it proved to be a TSPO-specific ligand [88]. This fluorinated radioligand **10** was also employed in a study to evaluate neuroinflammation in a mouse MS model (autoimmune encephalomyelitis, EAE): in vivo and ex vivo analyses showed that ^18^F-VC701 significantly accumulates within the CNS (cortex, striatum, hippocampus, cerebellum, and cervical spinal cord) of EAE compared to control mice, at 2 weeks post-immunization. The increase in ^18^F-VC701 uptake in EAE mice strongly evidenced the presence of microglia activation in the acute phase of the disease [89].

#### 2.2.2. 2-Phenylindolylglyoxylamides

The rational design of the 2-phenylindolylglyoxylamides (PIGAs) as TSPO ligands consisted in the reduction of the structural flexibility of the indole-3-acetamides, a series of derivatives synthesized by Kozikowski et al. endowed with high TSPO affinity and selectivity [90]. PIGAs exhibited a strong binding affinity for TSPO, ranging from nanomolar to subnanomolar levels [91,92,93]. Among them, the *N*^1^-methyl-2-(4′-nitrophenyl)indol-3-yl)glyoxylamide, [^11^C]NMPIGA (**11**, Figure 5, Table 1) showed remarkable affinity (*K*_i_ = 5.7 nM) and an acceptable lipophilicity (clog*P* = 3.95). When administered intravenously, **11** readily penetrated the monkey brain, resulting in a significant proportion of reversible specific TSPO binding. However, this novel chemical compound exhibited sensitivity to SNP rs6971. Specifically, **11** demonstrated similarly high affinities for HABs (*K*_i_ = 1.57 nM) and MABs (*K*_i_ = 1.82 nM) while displaying lower affinity for LABs (*K*_i_ = 9.53 nM) [43].

#### 2.2.3. Pyridazino[4,5-b]indole-5-acetamides

Pyridazino[4,5-*b*]indole-5-acetamides derived by the combination of the indole ring with a pyridazine one [44]. [^11^C]SSR180575, the 2-(7-chloro-5-methyl-4-oxo-3-phenyl-4,5-dihydro-3*H*-pyridazino[4,5-*b*]indol-1-yl)-*N*,*N*-dimethylacetamide (**12**, Figure 5, Table 1) was developed based on high affinity (*K*_i_ = 0.83 nM) and selectivity for TSPO of SSR180575, as well as the ability to support neuronal survival and regeneration in animal models of axotomy and neuropathy by facilitating the local synthesis of neurosteroids [44]; **12** exhibited substantial TSPO-specific binding, as demonstrated in a rodent model of acute neuroinflammation. In addition, increased uptake of **12** in the ipsilateral striatum, which exhibited elevated TSPO expression compared to intact tissue in the contralateral striatum, was also evidenced by in vitro autoradiography and in vivo brain PET imaging. Encouraging PET imaging properties of **12** were also observed in non-human primates [94,95].

After that, a series of related compounds was developed, by *N*^3^-position functionalization allowing for substitution with fluorine-18. Numerous compounds exhibited potent affinity, with *K*_i_ values similar to that of SSR180575. Among them, the derivative featuring a 3-fluoro-2-pyridyl moiety instead of the phenyl ring was selected for labeling, obtaining the 2-(7-chloro-3-(6-fluoropyridin-2-yl)-5-methyl-4-oxo-4,5-dihydro-3*H*-pyridazino[4,5-*b*]indol-1-yl)-*N*,*N*-dimethylacetamide, [^18^F]FPSSR180575 (**13**, Figure 5). In PET imaging evaluation using male Wistar rats bearing gliomas, **13** displayed increased accumulation in the tumor brain tissue relative to the contralateral non-tumor brain, thereby providing outstanding imaging contrast between the tumor and contralateral tissue [96].

#### 2.2.4. Phenoxyarylacetamides

**Figure 6:**
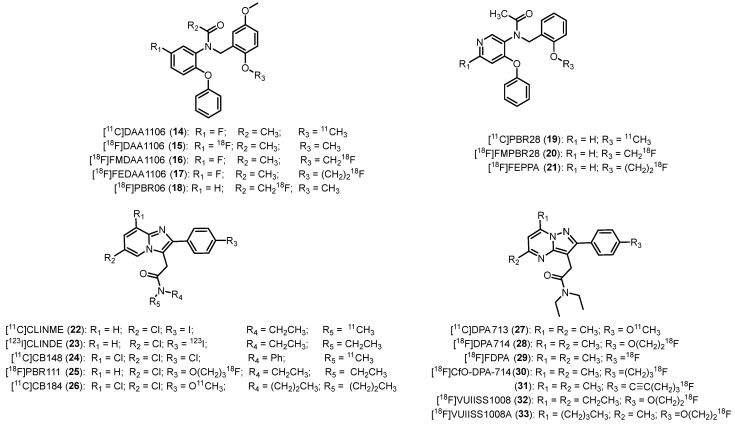
Second-generation TSPO radioligands.

Modification of Ro5-4864, involving the opening of the azepine ring, led to the generation of phenoxyarylacetamides as TSPO ligands. These compounds exhibit strong affinity and selectivity. Among them, the *N*-(2,5-dimethoxybenzyl)-*N*-(5-fluoro-2-phenoxyphenyl)acetamide, [^11^C]DAA1106 (**14**, Figure 6, Table 1) emerged as the first representative of this class to be radiolabeled [45,46]; **14** demonstrated 5-fold higher affinity and selectivity with respect to **1**; in addition, it resulted a species-independent radiotracer, as shown by its subnanomolar affinity in both rat and monkey brains (*K*_i_ = 0.043 nM and 0.188 nM, respectively). Furthermore, although it displayed a log*D* value slightly higher than the ideal range (log*D* = 3.65), it can cross the BBB [46]. Following its intravenous administration in mice, **14** assembled in regions of the brain with high TSPO density; **14** exhibited greater affinity for microglia compared to **1** in rat models of neuroinflammation [47]. In a study focused on the quantification of TSPO receptors in AD patients, **14** demonstrated heightened uptake in multiple brain regions of patients compared to age-matched healthy individuals [97]. Additionally, **14** was employed in PET studies involving patients affected by schizophrenia to investigate the role of glial cells in the disease’s pathophysiology [98]. It was also used in studies to confirm that smokers exhibit impaired inflammatory function in comparison to non-smokers [99].

In parallel with the development of **14**, efforts were made to synthesize the [^18^F]DAA1106 (**15**) and other ^18^F-labeled analogues, namely the *N*-(5-fluoro-2-phenoxyphenyl)-*N*-(2-(fluoromethoxy)-5-methoxybenzyl)acetamide, [^18^F]FMDAA1106 (**16**), and the *N*-(5-fluoro-2-phenoxyphenyl)-*N*-(2-(2-fluoroethoxy)-5-methoxybenzyl)acetamide, [^18^F]FEDAA1106 (**17**), Figure 6. Next, **16** was obtained by replacing a hydrogen atom in the methoxy group of **15** with fluorine, and **17** was obtained through the elongation of the methoxy chain by CH_2_ of **16** [100]. Biodistribution analysis of **15** demonstrated minimal radioactivity accumulation in bone, with no significant in vivo defluorination. Analysis of metabolites revealed that over 96% of the total radioactivity in the mouse brain, 60 min after the injection of the radiotracer, was in the form of unmetabolized **15** [101]; **16** showed almost similar affinity for TSPO as **15**, but it displayed significant absorption in monkey bones [102].

**17** exhibited high TSPO affinity with a *K*_i_ value of 0.078 nM, 2-fold higher than **15**. Moreover, **17** displayed elevated uptake in the monkey brain, particularly in the occipital cortex, with a radioactivity level 1.5 times higher than **15** [102]. However, a PET study involving control subjects and AD patients with such radioligand indicated that microglial activation does not be involved in this neurodegenerative disease [103].

In an effort to decrease metabolism and defluorination, the labeling position was changed, leading to the development of the [^18^F]*N*-fluoroacetyl derivative, namely the *N*-(2,5-dimethoxybenzyl)-2-fluoro-*N*-(2-phenoxyphenyl)acetamide, [^18^F]PBR06 (**18**, Figure 6) [104]. It demonstrated higher binding affinity than **1**, though interspecies variability existed: human tissue exhibited somewhat lower affinity (*K*_i_ = 1.0 nM) compared to monkey (*K*_i_ = 0.32 nM) and rat (*K*_i_ = 0.18 nM). Additionally, **18** displayed high lipophilicity (log*D* = 4.05) and over 90% of total uptake of specific binding in monkey brains. Human biodistribution analysis showed minimal defluorination with substantial radioactivity observed in metabolism and excretion-related organs, like liver and gallbladder. However, **18** has been extensively studied in various neurological disease models, including Huntington’s disease and stroke-associated neuroinflammation [105,106], showing to be an effective tool for characterizing microglial neuroinflammation over time in a mouse model of stroke [106]. [^18^F]PBR06 (**18**) is under study in various clinical trials that are currently recruiting patients; some trials have already been completed, while others are active but have not yet begun recruiting (https://clinicaltrials.gov/). The conditions investigated in these trials mainly include MS, Multiple System Atrophy (MSA), PD, AD, and myocardial infarction.

Phenoxypyridinylacetamides have also been developed as azaisosters of phenoxyphenylacetamides, which should possess a higher hydrophilicity and water solubility than their quite lipophilic counterpart. [^11^C]PBR28 (**19**), *N*-(2-methoxybenzyl)-*N*-(4-phenoxypyridin-3-yl)acetamide, displayed TSPO binding affinities similar to **18** (*K*_i_ = 0.68, 0.94, and 2.5 nM for rat, monkey, and human, respectively) but a more suitable lipophilicity (log*D* = 3.01), improved signal-to-noise ratio, and high specific binding in the brain (Figure 6, Table 1) [27,48]. This radioligand has been also employed in several studies in human subjects to study the progression of several diseases that involved microglia activation, including AD, MS, Huntington, motor neuron disease (MND), and rheumatoid arthritis diseases [32,73]. Unfortunately, this radioligand showed high sensitivity to the SNP rs6971. It was also recently used to evaluate whether chronic alcohol caused neuroinflammation [107]; **19** binding did not differ between alcohol use disorder (AUD) patients and healthy controls (HC). Nevertheless, when separating by TSPO genotype, MAB AUD participants showed lower **19** binding than HC, while no group differences were observed in HAB [50,108].

[^11^C]PBR28 (**19**) is being examined in various clinical trials that are currently recruiting patients; some trials have already been completed, while others are active but have not yet begun recruiting (https://clinicaltrials.gov/). The conditions investigated in these trials primarily include MS, PD, post-traumatic stress disorders (PTSD) and inflammatory arthritis.

Very recently, [^11^C]PBR28 (**19**) has been exploited to investigate seasonal changes in TSPO expression [109]. Despite limitations, such as high interindividual variability, test-retest inconsistency, tracer specificity, and a relatively small sample size, results indicate that TSPO levels in the brain are unaffected by light and temperature changes in different seasons [109].

Additionally, for this class of compounds, several efforts were made to synthesize ^18^F-labeled PBR28 analogues (Figure 6). The fluoromethoxy derivative, named the *N*-(2-(fluoromethoxy)benzyl)-*N*-(4-phenoxypyridin-3-yl)acetamide, [^18^F]FMPBR28 (**20**), showed an affinity measured by competition with [^3^H]PK11195 in a membrane of human leukocytes and a partition coefficient similar to those of PBR28 (IC_50_ = 8.28 ± 1.79 nM versus 8.07 ± 1.40 nM; log*D* = 2.85 vs. 2.82, new value determined by the authors) [110]. When directly compared in the same inflammatory rat model, **20** quickly reached the highest target-to-background ratio at early imaging time (35 min post injection vs. 85 min post injection for **19**), suggesting its promising use for imaging acute neuroinflammation. When employed in a rat model of experimental autoimmune myocarditis (EAM), **20** did not show a relevant difference in terms of specific TSPO-uptake between EAM and healthy rats [111]. The fluoroethoxy derivative [^18^F]FEPPA (**21**), *N*-(2-(2-fluoroethoxy)benzyl)-*N*-(4-phenoxypyridin-3-yl)acetamide, displayed higher affinity for TSPO with respect to **19** and **20** and suitable lipophilicity (log*P* = 2.99) (Figure 6, Table 1). In addition, it showed reduced metabolism and superior brain penetration in rat models [52]. When used for quantifying TSPO binding in the human brain, **21** emerged as a promising PET ligand for assessing neuroinflammation [112,113]. In details, when clinically tested, its binding was significantly higher in AD patients with respect to healthy ones in grey matter and in white matter regions [53]. In the same year, Setiawan et al. [114] evidenced high TSPO distribution volume (*V*_T_) during major depressive disorders in the grey matter areas. [^18^F]FEPPA was employed in several clinical trials to study several pathologies such as synucleopthies, psychosis/schizophrenia, depression and other disorders and to investigate neuroinflammation in participants with depression after the respiratory symptoms of coronavirus disease (COVID-19) have passed (https://clinicaltrials.gov/).

#### 2.2.5. Imidazo[1,2-a]pyridines

This class of compounds was designed as an analogue of alpidem, a compound endowed with nanomolar affinity for both TSPO and CBR [115]. [^11^C]CLINME (**22**, Figure 6), the 2-(6-chloro-2-(4-iodophenyl)imidazo[1,2-*a*]pyridin-3-yl)-*N*-ethyl-*N*-methylacetamide was the first compound of this class to be radiolabeled with carbon-11. It was used in acute rodent neuroinflammation, showing higher contrast between the TSPO expression in lesion site and that in the intact one with respect to **1** [116].

Subsequently, [^123^I]CLINDE (**23**), *N*’,*N*’-diethyl-6-chloro-(4′-[(^123^)I]iodophenyl)imidazo[1,2-*a*]pyridine-3-acetamide (Figure 6) has been characterized as SPECT tracer of inflammation, and its permeability across the BBB into the brain and TSPO specificity were showed in different animal models [117]. [^123^I]**23** uptake reflected astrogliosis in brain regions, including caudate putamen, corpus callosum, medium septum and olfactory tubercle [118]. Very recently, Ohshima et al. showed that [^123^I]CLINDE-SPECT imaging is effective for visualizing neuroinflammation and that the pattern of neuroinflammation differs according to the level of infarction severity [119].

[^11^C]CB148 (**24**, Figure 6, Table 1), the 2-(6,8-dichloro-2-(4-chlorophenyl)imidazo[1,2-*a*]pyridin-3-yl)-*N*-methyl-*N*-phenylacetamide represents an interesting alternative to **1**, showing subnanomolar TSPO affinity (*K*_i_ = 0.20 nM) and high accumulation in TSPO rich regions of the brain, with cerebellum and olfactory bulb (OB) that showed the highest uptake of **24** [55]. Only negligible amounts of metabolites were observed in brain tissue 30 min after intravenous injection of **24**.

PBR111, the (2-(6-chloro-2-(4-(3-fluoropropoxy)phenyl)imidazo[1,2-*a*]pyridin-3-yl)-*N*,*N*-diethylacetamide) is a metabolically stable imidazopyridineacetamide with high TSPO affinity (*K*_i_ = 3.7 nM) and selectivity and appropriate lipophilicity (Log*P* = 3.2) [120], rendering it suitable for imaging TSPO expression in neurodegenerative conditions [121]. [^18^F]PBR111 (**25**, Figure 6, Table 1) has undergone extensive evaluation in both preclinical experiments [56] and clinical trials to study pathologies such as AD and MS. However, it showed significant sensitivity to SNP rs6971, as confirmed in studies involving healthy individuals, where substantial differences have been observed among HABs, MABs, and LABs [122].

Furthermore, Van Camp and colleagues [123] conducted in vitro autoradiography studies and identified a substantial increase in the binding of **25** within areas of the rat brain subjected to alpha-amino-3-hydroxy-5-methyl-4-isoxazole propionic acid (AMPA) lesions in comparison to the control regions. Additionally, a heightened uptake of **25** in AMPA-lesioned rat brains compared to the uptake of **1** was observed through in vivo PET imaging.

[^11^C]CB184 (**26**) (*N*,*N*-di-*n*-propyl-2-[2-(4-methoxyphenyl)-6,8-dichloroimidazol[1,2-*a*]pyridine-3-yl] acetamide, Figure 6) represents another new candidate TSPO PET tracer endowed with subnanomolar TSPO affinity (*K*_i_ = 0.54 nM) and lower lipophilicity (log*P* = 2.06) with respect to **1** [124]. In mouse brain, **26** exhibited the highest uptake in regions such as the OB, cerebellum, hippocampus, and pons, with stability observed in uptake levels from 30 to 60 min post-injection. Pre-administration of **1** significantly reduced **26** uptake in all brain regions, underscoring its high binding specificity in mouse brain tissue. Metabolic analyses revealed that at 30 min post-injection, 92.7 ± 5.8% of **26** remained unchanged in the brain, and 36.2 ± 15.5% in the plasma [124].

Moreover, **26** PET imaging studies conducted in healthy volunteers revealed rapid brain uptake followed by swift clearance during a 90 min dynamic scan [125]; **26** demonstrated even distribution in gray matter, with the highest accumulation observed in the thalamus, closely followed by the cerebellar cortex and other regions.

#### 2.2.6. Pyrazolo[1,5-a]pyrimidines

This class of compounds was designed as bioisoster of imidazopyridines. The first compounds developed were the carbon-11-labeled [^11^C]DPA713 (**27**) (*N*,*N*-diethyl-2-(2-(4-methoxyphenyl)-5,7-dimethylpyrazolo[1,5-*a*]pyrimidin-3-yl)acetamide) (*K*_i_ = 4.7 nM) [57,58] and the fluorine-18 close analogue [^18^F]DPA714 (**28**) (*N*,*N*-diethyl-2-(2-(4-(2-fluoroethoxy)phenyl)-5,7-dimethylpyrazolo[1,5-*a*]pyrimidin-3-yl)acetamide) (*K*_i_ = 7.0 nM), Figure 6, Table 1 [59].

First, **27** showed a greater difference between healthy and diseased brain in parenchyma in an AMPA-induced rat model of neuroinflammation [58]. It possesses a better signal-to-noise ratio with respect to **1** due to its higher TSPO specific binding. Unfortunately, it suffers from SNP rs6971 sensitivity and radiometabolite accumulation in the human brain.

Next, **28** performed better than **27** in a rat model of acute neuroinflammation with the highest ratio of ipsilateral to contralateral uptake and the highest BP_ND_ [126,127]. In 2015, Lavisse and co-workers carried out a study on the pharmacological properties of **28** in the brains of monkeys, showing that [^18^F]DPA714 is concentrated in the hippocampus, occipital cortex, and the cerebellum [128]. Moreover, the degree of association of **28** with TSPO in the brain was approximately 73%, demonstrating the high specificity of the radioligand to the target in both normal and neurodegeneration-induced models. It has been successfully used for several purposes, such as (i) for image neuroinflammation in human models of AD and MS thanks to the enhanced uptake of [^18^F]DPA714 co-localized with infarct tissue [60,61]; (ii) to identify specific reactive areas of myeloid cell infiltration in a mouse model of orthotopic glioma in the tumor microenvironment [129]; and (iii) to monitor brown adipose tissue (BAT) activity in tumor-bearing mice in vivo [130]. [^18^F]DPA714 is under investigation in a number of clinical trials which are recruiting patients; several have been completed and a few are active but recruiting is yet to start (https://clinicaltrials.gov/). Conditions studied in these trials are primarily MS, PD, AD, tumors, myocardial infarction, and autoimmune encephalitis (AIE).

In a very recent study, Zhang et al. evidenced the potential value of ^18^F-DPA-714 PET tracer in supplementing magnetic resonance imaging (MRI) for AIE detection (clinical trial registration no. NCT05293405) [131].

With defluorination being the primary metabolic pathway in vivo for **28**, to overcome these problems, [^18^F]FDPA (**29**), the *N*,*N*-diethyl-2-(2-(4-fluorophenyl)-5,7-dimethylpyrazolo[1,5-*a*]pyrimidin-3-yl)acetamide (Figure 6, Table 1)—in which the fluorine atom was directly linked to the aromatic ring, a position that is expected to be resistant to defluorination—was developed [62]. Additionally, **29** exhibited high TSPO affinity (*K*_i_ = 1.7 nM) and appropriate lipophilicity (log*D* = 2.34) as well as the ability to easily cross BBB. In addition, the labeling position on the aromatic moiety imparts a higher stability with regard to in vivo metabolism [63]. In the APP/PS1 mouse model of AD, the radiotracer **29** exhibited an increase to 1.50 ± 0.13 SUV at 3 min post-injection, indicating a 1.6-fold higher uptake and slower washout when compared to age-matched control subjects. Keller et al. [132] demonstrated a substantial age-related elevation in **29** radioactivity in the brains of APP/PS1 mice. Notably, significant variations in binding between wildtype and transgenic animals in vivo at 9 months and ex vivo at 4.5 months were evidenced [132,133]. Moreover, [^18^F]FDPA can detect changes in TSPO expression after radiotherapy (RT) in head and neck squamous cell carcinoma (HNSCC) in mice. Although the physiological mechanisms behind this RT-induced uptake need to be further evaluated, the pharmacokinetic behavior of [^18^F]FDPA in HNSCC indicates this tracer to be suitable for TSPO imaging in cancer [134]. Despite these promising results, the transition of PET imaging with **29** to higher species has not been accomplished as of the current status of research.

With the same aim to overcome the metabolism problem associated to **28**, a small library of novel pyrazolo[1,5-*a*]pyrimidines featuring a fluoroalkyl or a fluoroalkynyl moiety at the *para*-position of the 2-phenyl ring was developed and biologically evaluated as TSPO ligands [135]. All derivatives displayed subnanomolar affinity (*K*_i_ = 0.37–0.86 nM) and high selectivity toward TSPO. The most active compounds, which also showed less sensitivity to microsomal metabolism and higher lipophilicity with respect to **28**, were radiolabeled with fluorine-18, leading to obtaining compounds **30** (*N*,*N*-diethyl-2-(2-(4-(3-fluoropropyl)phenyl)-5,7-dimethylpyrazolo[1,5-*a*]pyrimidin-3-yl)acetamide, known as CfO-DPA-714) and **31** (*N*,*N*-diethyl-2-(2-(4-(5-fluoropent-1-yn-1-yl)phenyl)-5,7-dimethylpyrazolo[1,5-*a*]pyrimidin-3-yl)acetamide), Figure 6. Both compounds were evaluated in vitro and in vivo for their TSPO specific binding and their potential as PET radiotracers in a rodent model of neuroinflammation. For both compounds, brain uptake and accumulation in the region affected by AMPA signaling validated their viability as PET radiotracers for in vivo applications [98].

The pyrazolopyrimidine scaffold was also subjected to optimization studies to explore the effect of the introduction of substituents with various steric bulk at the 5, 6, and 7- positions [136]. SAR analysis highlighted an enhancement in affinity achieved by substituting methyl with ethyl at the 5- and 7- positions of the pyrazolopyrimidine core. A derivative known as VUIIS1008 (2-(5,7-diethyl-2-(4-(2-fluoroethoxy)phenyl)pyrazolo[1,5-*a*]pyrimidin-3-yl)-*N*,*N*-diethylacetamide displayed subnanomolar affinity (*K*_i_ = 0.18 nM), representing a significant 36-fold improvement in binding affinity compared to **28**, Figure 6. In addition, this substitution displayed increased lipophilicity (log*P*_7_._5_ = 2.84 compared to 2.12 of **28**), a property deemed suitable for in vivo imaging. For these reasons, it was radiolabeled with fluorine-18 for imaging purposes. [^18^F]VUIIS1008 (**32**) showed rapid uptake in TSPO-rich organs. In preclinical PET studies involving both healthy mice and rats with gliomas, **32** demonstrated an enhanced tumor-to-background ratio and a higher BP_ND_ in tumors with respect to **28** [137]. Overall, these results highlighted **32** as a promising PET ligand for evaluating TSPO expression in glioma.

Further elongation of the alkyl chain at 7-position by the introduction of a *n*-butyl group led to derivative 2-(7-butyl-2-(4-(2-fluoroethoxy)phenyl)-5-methylpyrazolo[1,5-*a*]pyrimidin-3-yl)-*N*,*N*-diethylacetamide known as VUIIS1008A, which was radiolabeled with fluorine-18, leading to compound [^18^F]VUIIS1008A **33**, Figure 6 [138]. Such compounds showed picomolar TSPO affinity (IC_50_ = 16.2 pM), 700-fold higher than **28**. Unfortunately, the longer chain was also responsible for an increase in the lipophilicity of the molecule (log*D* = 3.7). However, compartment modeling of **33** demonstrated a greater tumor-to-background ratio, a higher tumor BP and a lower brain BP_ND_ with respect to **28** and **32**, thus suggesting **33** as a better candidate for imaging tumors with low TSPO expression [139].

#### 2.2.7. 2-Aryl-8-Oxodihydropurines

**Figure 7:**
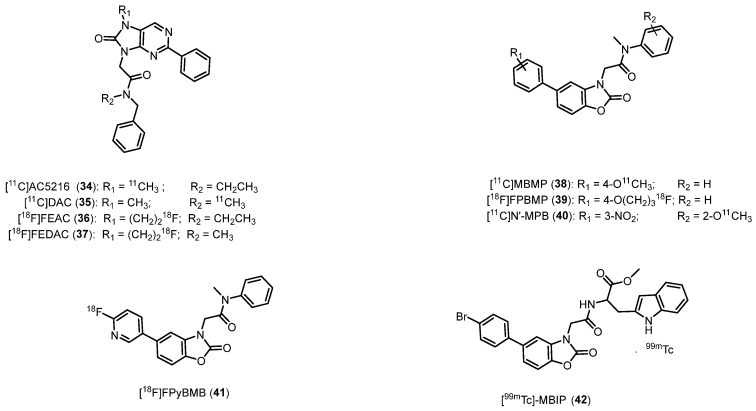
Second-generation TSPO radioligands.

In 1999 Murata et al. published a patent reporting a series of high TSPO affinity and selectivity ligands, namely the 2-aryl-8-oxodihydropurines (AOP) [140]. The most active compound, in terms of TSPO affinity (*K*_i_ = 0.2 nM) and suitable lipophilicity (log*D* = 3.3), was radiolabeled with carbon-11, obtaining the [^11^C]AC5216 (*N*-benzyl-*N*-ethyl-2-(7-methyl-8-oxo-2-phenyl-7,8-dihydro-9*H*-purin-9-yl)acetamide, **34**, Figure 7, Table 1) [64]. After injection of **34** into mice, a high radioactivity accumulation was observed in TSPO-rich organs, including lungs, heart, adrenal glands, OB and cerebellum. A PET study on monkey brain evidenced a relatively high uptake in the occipital cortex of **34**, an area characterized by high TSPO density in the primate brain. In addition, **34** showed high stability in vivo in the mouse brain [64]. It is of note that in a study performed to visualize microglial activation prompted by amyloid lesions in mouse models of AD, **34** facilitated PET imaging of glial TSPO with notable contrast, outperforming **17** in terms of effectiveness [141]. In 2009, the research group of Yanamoto developed a TSPO PET radioligand characterized by an oxopurine core, namely [^11^C]DAC (*N*-benzyl-*N*-methyl-2-(7-methyl-8-oxo-2-phenyl-7,8-dihydro-9*H*-purin-9-yl)acetamide) (**35**, Figure 7) [142]. It showed very similar affinity (*K*_i_ = 0.23 nM) for TSPO but lower lipophilicity (log*D* = 3.0) with respect to **34**. In normal mice, **35** displayed the highest radioactivity in the lung and a high level of radioactivity in the brain, evidencing its ability to cross the BBB and enter the brain. In vitro autoradiography and PET in rats subjected to kainic acid (KA)-induced lesions revealed, between **34** and **35**, similar levels of brain uptake in the lesioned and nonlesioned striatum. Of note, **35** exhibited a relevant increase of TSPO binding in the lesioned striatum. In addition, displacement experiments indicated high in vivo TSPO specific binding in the injured rat brain. Consequently, **35** proved to be a valuable PET radiotracer for TSPO imaging, and due to its TSPO specific binding, it stood out as a promising new biomarker for brain injury.

The same authors, in the following year, showed how high molar activity of **35** (average 4060 GBq/μmol) served as a valuable and sensitive biomarker for visualizing early infarction and characterizing the slightly elevated TSPO expression in the infarcted brain using PET [143,144].

The other two ^18^F-labeled oxopurines analogues—[^18^F]FEAC (**36**) (*N*-benzyl-*N*-ethyl-2-(7-(2-fluoroethyl)-8-oxo-2-phenyl-7,8-dihydro-9*H*-purin-9-yl)acetamide) and [^18^F]FEDAC (**37**) (*N*-benzyl-2-(7-(2-fluoroethyl)-8-oxo-2-phenyl-7,8-dihydro-9*H*-purin-9-yl)-*N*-methylacetamide)—were synthesized and evaluated as radioligands (Figure 7) [145,146]. Both compounds showed nanomolar affinity and good selectivity for TSPO (*K*_i_ = 0.49 and 1.34 for **36** and **37**, respectively). A small-animal PET scan conducted on a rat model of neuroinflammation revealed that **36** and **37** exhibited substantial accumulation of radioactivity in the KA-lesioned striatum. Both radioligands generated signals both in vitro and in vivo, enabling the visualization of the heightened TSPO expression in the rat brain following an infarct. The observed kinetics of both ^18^F-radiotracers in the monkey brain, along with their tolerance for in vivo metabolism, indicated their potential utility in imaging studies of TSPO in primates [147].

Further in vitro assays and in vivo PET conducted in activated macrophage and inflamed joints of collagen-induced arthritis (CIA) suggested the potential usefulness of **37** imaging in early stages of rheumatoid arthritis (RA) [148].

#### 2.2.8. Acetamidobenzoxazolones

Starting from 2012, a series of acetamidobenzoxazolones (ABOs) was reported as high-affinity TSPO ligands, designed as open analogues of Ro5-4864 [149,150]. The first radioligand of this class developed as a radiotracer for imaging purposes was [^11^C]MBMP (2-(5-(4-methoxyphenyl)-2-oxobenzo[*d*]oxazol-3(2*H*)-yl)-*N*-methyl-*N*-phenylacetamide **38**, Figure 7, Table 1) [65], selected for radiolabeling because of its nanomolar TSPO affinity (*K*_i_ = 0.28 nM) and adequate lipophilicity (Log*D* = 3.5) [65].

Although **38** demonstrated a higher BP_ND_ compared to **1** in the same ischemic model, it may not offer a real advantage over second-generation TSPO radiotracers. Notably, approximately 35% of radiolabeled metabolite from **38** was detected in the mouse brain 60 min post-injection, whereas this proportion was less than 10% for other TSPO radioligands in rodent brains [58,123], and this issue hampered its clinical application.

In the same year, the same research group developed the fluoropropyloxy analogue of **38**, the [^18^F]FPBMP (2-(5-(4-(3-fluoropropoxy)phenyl)-2-oxobenzo[d]oxazol-3(2*H*)-yl)-*N*-methyl-*N*-phenylacetamide, **39**, Figure 7) [69], which exhibited high stability, high affinity (*K*_i_ = 16.7 nM), equal lipophilicity (log*D* = 3.5) and TSPO specific binding in an ipsilateral ischemic rat brain in vitro.

Nevertheless, similar to numerous TSPO radiotracers, both **38** and **39** exhibited slow brain kinetics, associated with a lack of substantial reductions in radioactivity throughout the PET scan. This phenomenon was in part ascribed to their lipophilicity. In order to overcome this issue, a new TSPO radiotracer, namely [^11^C]N′-MPB (*N*-(2-methoxyphenyl)-*N*-methyl-2-(5-(3-nitrophenyl)-2-oxobenzo[*d*]oxazol-3(2*H*)-yl)acetamide, **40**, Figure 7, Table 1), characterized by the presence of a nitro group, was developed [66]; **40** displayed nanomolar TSPO affinity (*K*_i_ = 4.9 nM), reduced lipophilicity (log*D* = 2.08), and improved kinetic and metabolic stability than **38** in visualizing TSPO in a neuroinflammation model.

With the same aim of decreasing lipophilicity and, putatively, of obtaining more rapid brain kinetics, the same research group introduced a pyridine ring in the ABO scaffold, leading to obtaining [^18^F]FPyBMB (2-(5-(6-fluoropyridin-3-yl)-2-oxobenzo[*d*]oxazol-3(2*H*)-yl)-*N*-methyl-*N*-phenylacetamide, **41**, Figure 7) [151]. Such compound showed nanomolar affinity for TSPO (*K*_i_ = 13.4 nM) and modest lipophilicity (log*D* = 1.92). The higher TSPO binding of **41** on the ipsilateral side with respect to the contralateral one, and improved brain kinetics with respect to the other ABO radiotracers was evidenced by in vitro autoradiography and PET studies of ischemic rat brain. [151].

Finally, a TSPO ligand was developed by combining ABO and indole scaffolds, leading to a compound that was radiolabeled using technetium-99m [^99m^Tc] to be used as SPECT agent, [^99m^Tc]MBIP (methyl (2-(5-(4-bromophenyl)-2-oxobenzo[*d*]oxazol-3(2*H*)-yl)acetyl)tryptophanate, **42**, Figure 7). It displayed high stability in serum (>91%) and in solution (>94%) after 24 h. In addition, biodistribution analysis assayed on BALB/c mice evidenced significant accumulation of **42** in TSPO-rich organs and appropriate pharmacokinetic properties for a SPECT agent [152].

### 2.3. Third Generation TSPO Radioligands

#### 2.3.1. 2,3,4,9-Tetrahydrocarbazole-4-carboxamides

**Figure 8:**
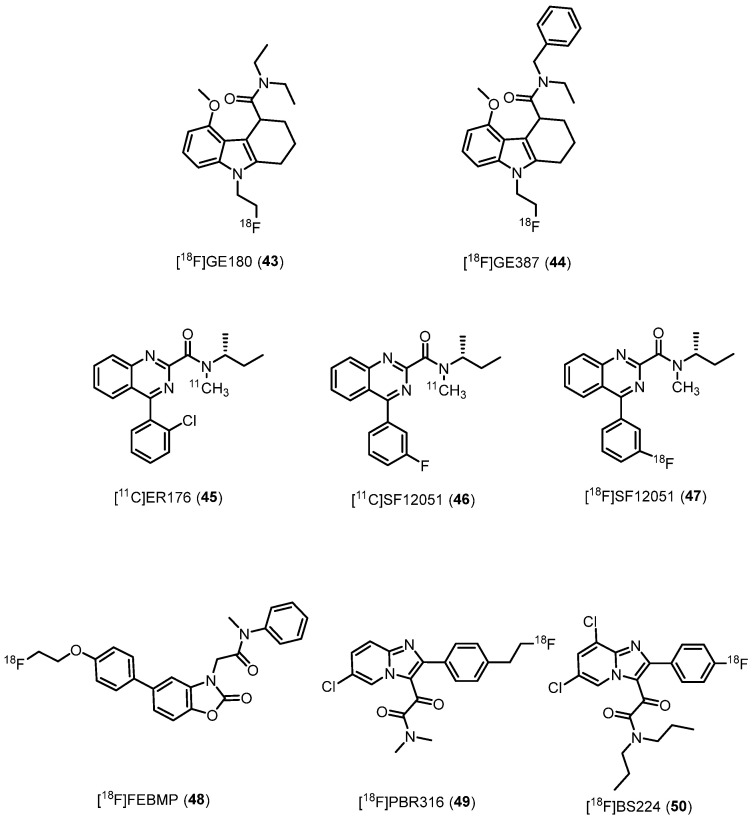
Third-generation TSPO radioligands.

Tetracyclic indole has been identified as a promising central core affording TSPO ligands [153]. From this pharmacophore, researchers created the tricyclic derivative *N*,*N*-diethyl-9-(2-fluoroethyl)-5-methoxy-2,3,4,9-tetrahydro-1*H*-carbazole-4-carboxamide [^18^F]GE180 (**43**, Figure 8, Table 1) [154]. The (*S*)-enantiomer exhibited higher affinity (*K*_i_ = 0.87 nM, rat; *K*_i_ = 9.2 nM, human) and binding specificity for TSPO-rich regions compared to the (*R*)-enantiomer (*K*_i_ = 3.87 nM, rat; *K*_i_ = 14.1 nM, human). Moreover, **43** demonstrated enantiomeric stability in vivo, making it a potential candidate for preclinical and clinical studies [67].

In preclinical models, **43** proved to be superior to **1** in imaging TSPO-associated neuroinflammation, displaying improved BP_ND_ and a longer half-life [155]. Its effectiveness was further demonstrated in studying neuroinflammation during normal aging and AD [156]. Moreover, in human studies, **43** showed low sensitivity to SNP rs6971 [157]; **43** was successfully used in patients with relapsing–remitting MS, detecting microglia/macrophage activation [158]. It also exhibited high tumor-to-background contrast in patients with glioblastoma [159]. Nonetheless, subsequent comparisons with **19** raised concerns about its brain penetration and signal-to-background ratio in diseases without a compromised BBB [160]. As a result, some research groups do not recommend its use [161]. Regardless, a number of clinical trials with **43** are active to study conditions such as AD, ischemic stroke, and glioblastoma (https://clinicaltrials.gov/).

Recently, [^18^F]GE387 (*N*-benzyl-*N*-ethyl-9-(2-fluoroethyl)-5-methoxy-2,3,4,9-tetrahydro-1*H*-carbazole-4-carboxamide, **44**, Figure 8) was developed, featuring a benzyl moiety instead of an ethyl substituent as in **43**. The (*S*)-enantiomer of **44** showed high TSPO affinity and demonstrated low sensitivity to SNP rs6971, similar to **1** [162]. PET scans in rats indicated its ability to enter the brain [163]. However, further biological evaluations of **44** are yet to be reported.

#### 2.3.2. Quinazolines-2-carboxamides

In 2012, the research group of Castellano developed a class of TSPO ligands as azaisoster of **1**, namely the quinazolines-2-carboxamides [164,165], that, due to their lower lipophilicity and higher water solubility, should display a better drug-like profile. Some of these quinazolines were chosen for their high affinity and suitable lipophilicity, radiolabeled with carbon-11 and subsequently assessed in monkey as PET radiotracers for visualizing TSPO in brain [25]. Of note, the direct azaisostere of PK11195, now dubbed [^11^C]ER176 (*N*-(*sec*-butyl)-4-(2-chlorophenyl)-*N*-methylquinazoline-2-carboxamide, **45**, Figure 8, Table 1), demonstrated higher affinity (*K*_i_ = 3.1 and 9.3 nM for **45** and **1**, respectively), lower lipophilicity (log*D* = 3.55 and 3.97 for **45** and **1**, respectively), higher TSPO-specific signal (>80%), higher proportion of TSPO-specific binding in monkey brain, and enhanced suitability for quantification without the presence of challenging radiometabolites with respect to **1**. Most importantly, **45** showed very low sensitivity to SNP rs6971 in vitro. In a following study [68], the same group in collaboration with the research group of V. W. Pike from the National Institute of Mental Health of Bethesda (Maryland) carried out whole-body imaging for nine human subjects from HAB, MAB, and LAB. Results evidenced a certain sensitivity of **45** to SNP rs6971 in vivo, in contrast to what was observed in in vitro studies. The reason behind this different sensitivity is unknown but, most probably, may be due to in vivo protein-protein interactions that do not occur in in vitro conditions. Nevertheless, **45** yielded sufficiently high BP_ND_ across all rs6971 genotypes, indicating that this radioligand is likely to exhibit greater sensitivity in detecting abnormalities in patients.

A straightforward and reliable HPLC separation technique has been developed for the automated production of [^11^C]ER176. The entire production process takes approximately 30 min and ensures effective separation from other radiochemical and chemical contaminants, allowing to obtain [^11^C]ER176 with high radiochemical and chemical purity, as well as excellent molar activity, meeting FDA current good manufacturing practice (CGMP) standards for clinical use [166]. [^11^C]ER176 is now subjected to clinical trials for neurodegenerative diseases, primarily AD (https://clinicaltrials.gov/).

From a comparison study between [^11^C]PBR28 **19** and **45**, the latter performed better showing a higher specific binding and a lower intersubject variability. Together, these results allowed the authors to conclude that such compounds are expected to hold greater statistical significance in clinical studies, necessitating a reduced number of subjects [167].

In a study aimed at quantifying TSPO in healthy humans, **45** demonstrated much greater specific binding than **1**. Furthermore, **45** exhibited a distinctive characteristic by not producing radiometabolites capable of entering the brain. For all these reasons, **45** is considered the most promising third generation TSPO radioligand for clinical research developed to date [21,28,73].

Recently [26], **45** analogues were designed by replacing the 2-chloro-phenyl ring with *o*-, *m*- and *p*- fluoro and trifluoromethylphenyl ones. All six compounds exhibited nanomolar affinity for TSPO (*K*_i_ between 1.2 and 7.0 nM) and low sensitivity to TSPO genotype in vitro. All six compounds were radiolabeled with carbon-11 and rapidly screened in mice, proving their ability to enter the brain and to provide a sizeable TSPO-specific signal. Among them, the *m*-fluoro analogue [^11^C]SF12051 (*N*-(*sec*-butyl)-4-(3-fluorophenyl)-*N*-methylquinazoline-2-carboxamide, **46**, Figure 8) displayed the most promising imaging behavior and for this reason was selected to be radiolabeled with fluorine-18, leading to obtaining [^18^F]SF12051 (**47**, Figure 8). Preliminary imaging study evidenced that **47** gave comparably promising results in the mouse brain to that of **46**.

In vivo studies in the monkey brain of all the six ^11^C-labeled analogues were also performed. The two in vivo criteria taken into consideration were the BP_ND_ and the time stability of total *V*_T_, an indirect measure of lack of radiometabolite accumulation in the brain [168]. Results showed that all six ^11^C-labeled analogues had a good BP_ND_ and good time stability of *V*_T_. Among them, the *o*-fluoro, *m*-trifluoromethyl, and *m*-fluoro derivatives resulted in the three best candidates being labeled with fluorine-18.

Other third-generation ligands. [^18^F]FEBMP (2-(5-(4-(2-(fluoro-^18^F)ethoxy)phenyl)-2-oxobenzo[*d*]oxazol-3(2*H*)-yl)-*N*-methyl-*N*-phenylacetamide, **48**, Figure 8, Table 1), belonging to the ABO family (see Section 2.2.8.) developed by Tiwari et al. [69], displayed high stability, TSPO nanomolar affinity (*K*_i_ = 6.6 nM), appropriate lipophilicity (log*D* = 3.4), and TSPO-specific binding in an ipsilateral ischemic rat brain in vivo. Most importantly, **48** showed low sensitivity to SNP rs6971, as observed in in vitro autoradiography on postmortem human brains [169]. It is still to be understood which is the suitable chemical structure for showing binding to LAB. In fact, even a slight modification in the side chain might greatly affect the binding affinity for LAB. Actually, given a certain core structure such as that of the phenoxyphenylacetamides, the ratio of *K*_i_ for LAB to HAB (R_Ki(L/H)_) of [^11^C]PBR28, [^18^F]PBR06, and [^11^C]DAA1106 is greatly different, with values of 55, 17, and 4.7, respectively [19]. Thus, given that the prevalence of Thr147 (low-binding allele) is 30% in Caucasians and 25% in Africans [23], [^18^F]FEBMP could have a general utility in the sensitive detection of neuroinflammation. Furthermore, the authors conclude, [^18^F]FEBMP could serve as a core chemical structure for new PET ligands in the entire population, including in individuals with TSPO rs6971 polymorphisms.

[^18^F]PBR316 (2-(6-chloro-2-(4-(2-(fluoro)ethyl)phenyl)imidazo[1,2-*a*]pyridin-3-yl)-*N*,*N*-dimethyl-2-oxoacetamide, **49**, Figure 8, Table 1) belongs to the imidazo[1,2-*a*]pyridine family. It showed nanomolar affinity (*K*_i_ = 6.0 nM) and high selectivity, low sensitivity to SNP rs6971 with a LAB/HAB ratio of 1.5 [70]. Biodistribution in rats of **49** showed a high uptake in organs known to express TSPO such as heart (3.9%) and adrenal glands (7.5% ID per g) at 1 h. [^18^F]PBR316 entered the brain and accumulated in TSPO-expressing regions and radioactivity was blocked by PK11195 and Ro5-4864 but not flumazenil. Metabolite analysis indicated that radioactivity in adrenal glands and the brain was predominantly due to the intact radiotracer. Due to these characteristics, [^18^F]PBR316 **49** appears suitable for further biological and clinical studies.

[^18^F]BS224 (**50**, Figure 8, Table 1), 2-(-2-(4-fuorophenyl)-6,8-dichloroimidazo[1,2-*a*]pyridin-3-yl)-*N*,*N*-dipropylacetamide, is the newest ^18^F-labeled imidazo[1,2-a]pyridine TSPO radiotracer, endowed with high affinity (*K*_i_ = 0.51 nM) and selectivity, suitable lipophilicity (Log*D* = 2.78),  and low sensitivity to SNP rs6971 (RKi(L/H) = 0.76) [71]. Docking studies confirmed that the A147T mutation did not affect the binding mode of **50**, supporting the observed in vitro TSPO selectivity of **50** regardless of polymorphisms. PET imaging findings indicated that **50** exhibited a notable target-to-background ratio in the initial imaging stages of brain regions affected by abnormalities in TSPO expression. It enabled a distinct and visible representation of inflammatory lesions in both animal models (LPS-induced inflammatory and ischemic stroke rat models), devoid of interference from skull uptake [71]. Based on these results, authors asserted that [^18^F]BS224 **50** might be a promising next-generation TSPO PET ligand to evaluate neuroinflammatory disease-relevant areas in an ample array of patients irrespective of the common rs6971 polymorphism.

Third-generation TSPO radioligands were developed to have adequately high BP_ND_ across all *rs6971* genotypes for reliable quantification and to lack the brain radiometabolite accumulation that interferes with the low specific signal in LABs. However, while these novel third-generation ligands may offer improvements over second-generation ones, they still exhibit sensitivity to the A147T variant of TSPO. To make further progress, it is crucial to deepen our understanding of the differing binding requirements between WT and A147T TSPO. As ^11^C-ER176 is the most promising third-generation TSPO radioligand for clinical research [28,32,168] displaying high specific binding (>80%), an adequately high BP_ND_ in LABs, good time stability of total distribution volume (*V*_T_) across all genotypes, and no significant amount of radiometabolites accumulating in the brain, future research aimed at resolving the human TSPO crystal structure in complex with this third generation ligand will be crucial in understanding the different binding requirements of WT and A147T TSPO, thus speeding up the development of TSPO PET radioligands that are not affected by the rs6971 genotype [33]. Moreover, it will be essential to investigate the brain permeability and radiometabolite production of identified lead molecules to ensure that radiometabolites do not interfere with the analysis of brain signals. Other challenges to face will be with respect to pharmacokinetics, bioavailability, and safety.

## 3. Conclusions

TSPO is broadly spread through the whole body, with the highest concentration in tissues that synthesize steroids. A significant increase in TSPO is linked to microglial activation in response to brain injury and neuroinflammation. Consequently, heightened TSPO expression is regarded as a key indicator of neuroinflammation. In this context, TSPO radiotracers have been recognized as potent tools for imaging neuroinflammation.

Despite various challenges related to quantification, allelic binding dependence, microglial phenotype, and incomplete cellular specificity for microglia, TSPO PET imaging of neuroinflammation has remained a focal point in studies of neuroinflammatory diseases. The data collected in this review corroborate the huge efforts in developing more specific tracers during the last forty years.

TSPO PET radiotracers have been classified in three generations. First-generation TSPO radioligands include the isoquinoline carboxamide [^11^C](*R*)-PK11195 (**1**) and the benzodiazepine [^11^C]Ro5-4864 (**2**). Even if **1** is still today the most widely used radiotracer for in vivo imaging of TSPO, it suffers from several drawbacks, such as a high level of nonspecific binding and a signal-to-noise ratio that is not optimal, complicating its quantification. Second-generation probes belong to different structural classes endowed with better properties such as enhanced affinity, reduced lipophilicity, improved TSPO-specific signals, as well as superior imaging properties. However, these radioligands suffer from sensitivity to SNP rs6971. Finally, third-generation radiotracers overcame almost all of the limits associated with the second generation and yielded superior outcomes, particularly in preclinical and clinical investigations.

Although these advances in the discovery of less-discriminating TSPO PET ligands, the development of truly non-discriminating ligands will require a better knowledge of the differences characterizing the A147T and WT TSPO structures, and the development of structure–activity relationships through high-throughput screening or artificial intelligence methods such as deep and machine learning that, in the last years, has transformed the approach to drug discovery task by delivering added value in small-molecule drug discovery.

In conclusion, this review aims to offer a perspective on the sequential progress achieved in the development of TSPO PET probes during the past four decades. It also seeks to provide an overview of the currently accessible radioligands for neuroinflammation imaging both in research and in routine clinical settings. This information may assist (radio)chemists in the future design and development of more specific and efficient probes. Indeed, the ongoing advancement of PET radioligands for neuroinflammation imaging presents an appealing and noninvasive method, offering the potential for early diagnosis, disease progression monitoring, and contributing to the rational design and clinical evaluation of patient responses to therapeutic interventions.

## Figures and Tables

**Table 1:** The most representative compounds in PET imaging of neuroinflammation.

Generation	Chemical Class	Representative Compounds	*K*_i_ (nM)	PET Imaging Study in Human Pathologies	Refs
1st	Isoquinoline carboxamides	[^11^C]-(*R*)-PK11195 (**1**) [^11^C]-(*S*)-PK11195 (**1**)	9.0 19	MCI, AD, PDD, MSA, PSP, CBD, FTD	[27,39]
	Benzodiazepines	[^11^C]Ro5-4864 (**2**)	6.0	HG	[40]
2nd	Quinoline-2-carboxamides	[^11^C]VC195 (**8**)	2.1	-	[41]
		[^11^C]VC701 (**9**)	0.11	-	[42]
	2-Phenylindolylglyoxylamides	[^11^C]NMPIGA (**11**)	5.7	-	[43]
	Pyridazino[4,5-b]indole-5-acetamides	[^11^C]SSR180575 (**12**)	0.83	-	[44]
	Phenoxyarylacetamides	[^11^C]DAA1106 (**14**)	0.043	MCI and AD	[45,46,47]
	Phenoxypyridinylacetamides	[^11^C]PBR28 (**19**)	0.68	MS, TLE, MCI, AD, PCA, HD and ALS	[48,49,50,51]
		[^18^F]FEPPA (**21**)	0.07	AD, MDD, OCD and PD	[52,53,54]
	Imidazo[1,2-a]pyridines	[^11^C]CB148 (**24**)	0.20	-	[55]
		[^18^F]PBR111 (**25**)	3.7	healthy	[56]
	Pyrazolo[1,5-a]pyrimidines	[^11^C]DPA713 (**27**)	4.7	TLE, PD and AD	[57,58]
		[^18^F]DPA714 (**28**)	7.0	AD and MS	[59,60,61]
		[^18^F]FDPA (**29**)	1.7	-	[62,63]
	2-Aryl-8-oxodihydropurines	[^11^C]AC5216 (**34**)	0.2	healthy	[64]
	Acetamidobenzoxazolones	[^11^C]MBMP (**38**)	0.28	Ischemic brain	[65]
		[^11^C]N’-MPB (**40**)	4.9	-	[66]
3rd	Tetrahydrocarbazole-4-carboxamides	[^18^F]GE180 (**43**)	0.87	RRMS	[67]
	Quinazolines-2-carboxamides	[^11^C]ER176 (**45**)	3.1	healthy	[25,68]
	Other ligands	[^18^F]FEBMP (**48**)	6.6	-	[69]
		[^18^F]PBR316 (**49**)	6.0	-	[70]
		[^18^F]BS224 (**50**)	0.51	-	[71]

Alzheimer’s disease (AD), amyotrophic lateral sclerosis (ALS), corticobasal degeneration (CBD), frontotemporal dementia (FTD), Huntington’s disease (HD), human glioma (HG), mild cognitive impairment (MCI), major depressive disorder (MDD), multiple sclerosis (MS), multiple system atrophy (MSA), obsessive compulsive disorder (OCD), posterior cortical atrophy (PCA), Parkinson’s disease (PD), PD dementia (PDD), progressive supranuclear palsy (PSP), elapsing remitting multiple sclerosis (RRMS), and temporal lobe epilepsy (TLE).

## Data Availability

Not applicable.
